# A qualitative study examining transgender people’s attitudes towards having a child to whom they are genetically related and pursuing fertility treatments in Greece

**DOI:** 10.1186/s12889-021-10422-7

**Published:** 2021-02-18

**Authors:** P. Voultsos, C.-E. Zymvragou, M.-V. Karakasi, P. Pavlidis

**Affiliations:** 1 Laboratory of Forensic Medicine & Toxicology (Medical Law and Ethics), School of Medicine, Faculty of Health Sciences, Aristotle University of Thessaloniki, University Campus, GR 54 124 Thessaloniki, Greece; 2 Laboratory of Forensic Sciences, Democritus University of Thrace, School of Medicine, Dragana, GR 68100 Alexandroupolis, Greece

**Keywords:** Transgender people, Fertility preservation, Assisted reproductive technologies, Discrimination, Fertility counseling, Transgender parenthood, Greece

## Abstract

**Background:**

Advances in biomedical technologies permit transgender individuals not only to achieve gender transition but also to experience parenthood. Little is known about this topic in Greece, which, although a traditionally conservative country, is changing at the legal level towards a greater recognition of transgender people’s rights. This study aimed to investigate transgender people’s attitudes towards having a child to whom they are genetically related and pursuing fertility treatments in Greece.

**Methods:**

This is a prospective qualitative study conducted with adult individuals who identified as transgender men or transgender women between April 2019 and March 2020. Individual in-depth qualitative interviews were conducted with 12 participants. The interviews were carried out in person and were digitally recorded and transcribed verbatim. We performed a thematic analysis of the data.

**Results:**

The thematic data analysis resulted in the identification of themes that represent key barriers to pursuing fertility preservation or the use of assisted reproductive technology. Six major themes were clearly present in the findings (lack of adequate information and counseling, worsening gender dysphoria, increased discrimination against transgender people due to the rise of extreme far-right populism, low parental self-efficacy, high costs, and a less-than-perfect legal framework). Moreover, diverse cases were examined, and minor themes, such as the symbolic value of the uterus and pregnancy, the relationship between the type of gender transition and willingness to pursue fertility treatments, and transgender people’s adherence to heteronormative patterns in the context of reproduction, were identified. Various reasons for transgender people’s differing degrees of desire for parenthood were identified.

**Conclusion:**

Our findings demonstrated contextual factors as well as factors related to transgender people themselves as barriers to pursuing transgender parenthood. Most aspects of our findings are consistent with those of previous research. However, some aspects of our findings (regarding aggressive behaviors and economic instability) are specific to the context of Greece, which is characterized by the rise of extreme far-right populism due to the decade-long Greek economic crisis and a deeply conservative traditionalist background. In that regard, the participants highlighted the (perceived as) less-than-perfect Greek legislation on transgender people’s rights as a barrier to transgender (biological) parenthood.

**Supplementary Information:**

The online version contains supplementary material available at 10.1186/s12889-021-10422-7.

## Background

An increasing number of young transgender people today are using medical procedures such as gender-affirming hormonal or surgical therapies to achieve gender transition^1^ [1, 2]. Gender transitioning is ‘the process of changing one’s gender presentation and/or sex characteristics to accord with their internal sense of gender identity’ [3]. Importantly, in the past, young transgender people never sought gender-affirming care (i.e., hormonal) as part of the transition process at earlier stages of development [1]. While research has shown that gender-transitioning people experience psychological benefits [4], the multifaceted process of gender transitioning with hormones or sex reassignment surgery may introduce a higher risk of significant long-term implications, including temporary or permanent loss of fertility [5, 6]. Notwithstanding, recent advances in biomedical technologies have not only enabled gender transition but also made it feasible for transgender individuals to experience parenthood. Most transgender people who become parents do so through biological means [7]. At present, fertility preservation (FP) techniques include sperm banking for transgender women and oocyte, embryo, or ovarian tissue banking for transgender men, while new FP techniques may be developed in the future. For instance, uterus transplantation may become available in the future (although not the foreseeable future) for transgender women.

Consequently, transgender people face complex and difficult decisions about whether to freeze sperm or eggs or use assisted reproductive technology (ART) [6]. The introduction of alternative means of achieving biological parenthood through medical advances has, therefore, created new forms of families including (at least) one transgender person. However, ‘the uptake of this option to date has been low’ [8]. A few years ago, the academic literature suggested that little was known ‘about how transgender people create their families and the issues involved in these decisions’ [9]. More specifically, it was stated that ‘little is known about their desire to have children and attitudes towards fertility preservation options’ [10]. Moreover, it was argued that because there was little knowledge about the complex topic of ‘medically assisted reproduction among transgender people’, more clarification was needed [11]. However, there is now a substantive body of research on the creation of families by trans people, and there has been a significant increase in research on FP over the past few years [7, 12–19]. Recently, Sterling and Garcia conducted a systematic literature search of PubMed, Medline and Google Scholar and identified several publications related to the topic of interest [20].

This manuscript attempts to expand knowledge about transgender adults’ attitudes and desires related to family formation and FP in Greece, as further empirical research is needed to provide a more nuanced exploration of transgender people’s rights, including their right to equal access to healthcare services [21]. There is a lack of empirical evidence to support an understanding of what it is like for transgender people in Greece to make a decision about whether to pursue FP or ART. Greek society is traditionally conservative. However, within the recently changing legal framework that greatly strengthened transgender rights by allowing citizens to choose to legally change their gender identity, more transgender people are expected to use fertility clinics. If this is the case, fertility clinics will face an entirely new patient group (transgender people) ‘whose reproductive futures were previously considered either impossible or undesirable [and] are now “anticipating infertility” and engaging in “family planning” as central parts of their lifecourse and medical engagements’, as Payne and Erbenius (2018) wrote with respect to Sweden [22].

### The legal status of transgender people in Greece

Since 2013, the Greek Criminal Code has punished gender identity discrimination and violence, and this legal protection was enhanced by the anti-racism law, Law n.4285/2014. Nevertheless, over recent years, Greece has adopted extreme austerity measures that have led to the rise of far-right parties. Consequently, homophobic and transphobic physical and rhetorical violence have substantially increased [23, 24]. More recently, Law n. 4491/2017 allowed citizens to choose to legally change their gender identity (from the age of 15). Importantly, this law improved transgender people’s right to change their official gender registration according to their own understanding of their gender identity without requiring gender-affirming treatment. Under the new law, young people (between the ages of 15 and 17) can apply for legal changes in their gender identity after having obtained a certificate issued by a medical council (in Athens Children Hospital). The law brings Greek legislation in line with the legislation of most EU countries [25], and Transgender Europe (2017) welcomed this law [26]. Undoubtedly, this law is an important step in improving transgender people’s autonomy. Note, however, that transgender people who already have children when they apply for a legal change to their gender identity are presented on the registry certificates of their children according to their former gender identity (their sex assigned at birth). As a consequence, the current legal framework ‘prevents’ transgender parents from applying for legal changes to their gender identity.

Unsurprisingly, legal amendments can hardly alter issues that are rooted in culture [24]. The Orthodox Church of Greece has profoundly shaped Greek people’s moral and social attitudes for many years. The Orthodox Church of Greece stated that the Law n. 4491/2017 was ‘a satanic deed’ that will lead to ‘the destruction of social cohesion…’ [25]. Greek cultural values place considerable emphasis on heterosexual coupledom, promoting the view that it is a prerequisite for personal fulfillment [27]. Religion is a major factor that strongly influences Greek culture, particularly regarding sexuality and marriage.

In Greece, Laws n.3089/2002 and n.3305/2005 constitute a regulatory environment that is largely liberal compared to those of many other European countries and that allows citizens to access in vitro fertilization (IVF) techniques such as heterologous fecundation (assisted fertilization of a woman’s oocyte with donor sperm), surrogacy, postmortem fertilization, cryopreservation and donation of gametes or zygotes. Under the current Greek legal framework, IVF is permitted only for strictly medical reasons, namely, for individuals ‘unable to have children naturally’ (Greek Civil Code, article 1455§1). Hence, access to IVF techniques is not granted to same-sex couples or single men. However, a lesbian trans woman can access IVF techniques by presenting herself as a ‘single woman’ wanting a child (Law n. 3089/2002 in combination with Law 4491/2017). ‘Trans women can opt for semen cryopreservation prior to their gender-affirming transition to retain the possibility to parent genetically related offspring’ [28]. Trans women may seek surrogacy to achieve genetic parenthood.

### Methodological aspects

#### Instrument

The present work is a prospective qualitative research study centered on exploring the social realities of individuals who identify as transgender and their descriptions of their lived experiences and attitudes towards having biological offspring. Data were collected through semistructured in-depth interviews conducted in person with 12 individuals who identified as transgender men or transgender women between April 2019 and March 2020.

#### Research questions

The primary research question that defined the focus of this study was as follows:

What are the attitudes of adult transgender women and transgender men towards having a child to whom they are genetically related and pursuing fertility treatments in Greece?

The secondary research questions were as follows:

- What are the factors (if any) affecting transgender individuals’ fertility decisions?
- What are the challenges (if any) that transgender people face in accessing fertility treatment or pregnancy and birth services?

We followed each of the items listed in the COREQ (COnsolidated criteria for REporting Qualitative research) checklist [29].

### Research team and reflexivity

#### Personal characteristics

C-E Z conducted the interviews. She is a psychologist who was pursuing a master’s in bioethics at the time of the study and has experience in conducting qualitative research interviews. PV is an Associate Professor of Medical Ethics, V-M K is a physician (psychiatry resident), and PP is an Associate Professor of Forensic Medicine.

#### Relationship with participants

No relationships between the interviewer and participants were established prior to study commencement. The interviewer’s reasons for doing the research as well as her interest in the research topic were reported to the participants.

### Study design

#### Theoretical framework

Thematic analysis (a widely used qualitative research technique) was selected as the methodological orientation to underpin the study.

#### Participant selection

Purposive sampling was used to deliberately identify individuals who identified as transgender persons and potentially had experience with transgender parenthood and fertility treatment. Purposive sampling was used to select individuals willing to provide detailed information about their perceptions, attitudes and experiences of having biological offspring and pursuing FP and/or in vitro fertilization techniques. The participants represented a wide range of ages and diverse socioeconomic backgrounds. Initially, we approached people who identified as transgender (but not nonbinary) persons using the interviewer’s (C-E Z) personal contacts. Overall, 12 participants were recruited through community outreach and the interviewer’s personal contacts. Potential participants were approached in person, by phone or by email and then contacted by phone to schedule an interview. None of the potential participants refused to participate or dropped out. Recruitment continued from April 2019 through March 2020, reaching a total of 12 participants. After first contact, all of the individuals were told that the purpose of the study was to understand the attitudes of trans people towards undergoing FP and having biological offspring in Greece and that the interview was expected to take between 30 and 60 min to complete. After agreeing to participate, the participants received a brief explanation of the objectives and the policies regarding anonymity, voluntary participation and confidentiality of the study. All interviews were conducted in Greek.

#### Setting

The interviews were conducted in neutral places of the participant’s choice. All interviews were held in quiet places (most often private rooms) with a comfortable environment. As phenomenological researchers, we were interested in describing the participants’ experiences while maintaining a natural (normal, unreflective and effortless) attitude. No one aside from the participant and interviewer was present at the interviews.

#### Description of the sample

The selected study participants (*N* = 12) were individuals who identified as transgender men and women and were in different stages of transition; they were diverse in terms of age, gender identity, transition phase or type, place of residence, sexual orientation, and educational background. The age of the participants ranged from 23 to 60 years, with the majority being between 27 and 45. The mean (standard deviation, SD) age of the participants was 40 (11) years. All participants were adults and had been Greek citizens for at least the last 10 years. All participants resided in urban areas. The participant characteristics are presented analytically in Table 1.

**Table 1:** Demographic items: counts and percentages

Variable	
**Age (years)**	
< 30	4 (33%)
30–50	5 (42%)
> 50	3 (25%)
Mean (SD)	40 (11)
Minimum–maximum	23–60
**Self-reported gender identity**	
Trans man	8(66%)
Trans woman	4(34%)
**Place of residence**	
Athens	3(25%)
Thessaloniki	4 (33%)
Other	5 (42%) [including Northern Greece and Crete; one was from Cyprus]
**Type of transition**	
Medical	10 (83%)
Social	2 (17%)
**Stage of the transition process**	
Incomplete	9 (75%)
Complete	3 (25%)
**Children**	
Has children	1(8,3%), has 3 children from previous relationship
**Education**	
Less than high school	None
High school graduate	10 (83%)
Post-high school education	2 (17%)
**Sexual orientation**	
Heterosexual/Straight	10 (83,3%)
Homosexual/Gay	0
Bisexual	1 (8,3%)
Pansexual	1(8,3%)
**Sex work**	
Yes	1(8,3%)
No	11(91,7%)

#### Data collection

The interviews were conducted one on one. The interview guide was developed based on a review of the relevant literature and then, as a first step, pilot tested. The guide was slightly refined based on the initial results from a few interviews to help the participants to better understand the specific issues being asked about in the questions. We next developed an informal grouping of topics and questions that the interviewer could ask in different ways for different participants. The interview guide covered a number of topics to capture a wide range of the participants’ lived experiences. These topics were related to a) making fertility decisions and b) accessing fertility treatment and health care services. The participants were encouraged to expand upon the examined topics. They were asked broad questions and encouraged to respond in a conversational way to express themselves. The interviews were semistructured and started with questions such as “What was it like to be a transgender parent, and what does it mean to you?” (*a grand tour question to make the participant comfortable*), “How do you think other transgender people perceive having a child to whom they are genetically related?”, “What would motivate or did motivate you to pursue or not pursue parenthood?”, “What do you know about other transgender people’s experiences or attitudes towards pursuing fertility preservation or in vitro fertilization techniques?”, and “Can you please describe in detail what types of barriers a transgender person needs to overcome to pursue fertility preservation or in vitro fertilization techniques?”. The set of interview guide questions is presented in [Additional file 1] (Supplementary Material). Additional questions were asked to elicit more detailed explanations and identify the essential themes of transgender people’s attitudes towards having a child to whom they are genetically related and pursuing fertility treatments.

We did not carry out follow-up interviews. The interviewer audio-recorded the interviews to collect the data. In addition, field notes were made after the interview to record nonverbal behavior patterns, as well as procedural and contextual aspects of the interviews, which enabled deeper and contextual critical reflection on the data collected. The interviews lasted from 38 min to 55 min each (mean 44 min). They were digitally audio-recorded and transcribed verbatim to preserve authenticity. We stopped data collection when we believed data saturation had been reached, namely, when no additional information was obtained from further interviews. The interview transcripts were not returned to the participants for their comments and/or corrections.

#### Data analysis

The research data were gathered by combining conversational interviewing and structured interviewing to yield insightful findings. The interviewer spent the first part of the interview gaining the participants’ trust. For this reason, in all the interviews, the initial rapport-building was devoted to addressing the apprehension phase of the interview process [30]. This phase was largely devoted to discussing topics not directly related to the research topic, such as gender dysphoria, social stigma and discrimination, and the gender transitioning process. Interestingly, this part of the interviews was found to be useful for improving the data interpretation in the thematic analysis.

Qualitative data were analyzed using thematic analysis [31]. As we wanted to work with our research participants as collaborators, examining their different perspectives, we used the flexible method of thematic analysis, which can generate unanticipated insights [31]. As transgender men’s experiences of barriers in making fertility decisions or accessing fertility treatment or pregnancy and birth services had not been previously explored in the context of Greece, we were not already aware of the participants’ probable responses. We followed Gibbs’ (2007) [32] advice on demonstrating qualitative reliability. Furthermore, thematic analysis was conducted to produce trustworthy and insightful findings and “to make sense of the data, and tell the reader what it does or might mean” [33].

Verbatim transcription of the audio-recorded narratives was performed. In the first step, we read through the entire data set at least once to familiarize ourselves with all aspects of the interview data, capture initial thoughts and take notes before beginning coding. In a second step, we identified important sections of text and attached initial codes to indicate them as related to themes in the data. While generating the initial codes, we highlighted similarities and differences in the perspectives of different research participants. Then, in a third step, we maintained detailed notes about the hierarchies of themes to be included in the devised set of themes. In a fourth step, we reviewed the themes and, more specifically, the coherence between the coded data extracts. We checked whether there was some overlap between themes and whether some themes might need to be broken down into separate themes [33]. In the fifth step, we defined and named themes, writing a detailed analysis of each one individually. It should be highlighted that we allowed sufficient time for all of the data to be read through and the coding to be reviewed at least twice. Moreover, we coordinated communication and shared analyses. We strived to capture and investigate in depth all aspects of the participants’ narratives related to the research goal.

A data management software program (NVIVO, 2015) was used to manage the data, namely, to secure and further refine the systematic character of the analysis. The participants did not provide feedback on the findings. Participant quotations are presented to illustrate the themes and findings. Each quotation is identified with the pseudonym of the participant. There is consistency between the data presented and the findings. Five major themes were clearly identified in the findings (lack of adequate information and counseling, worsening gender dysphoria, increased discrimination against transgender people due to the rise of extreme far-right populism, low parental self-efficacy, and high costs). Moreover, diverse cases are described, and minor themes (such as the symbolic value of the uterus and pregnancy, the relationship between the type of gender transition and the willingness to pursue FP and IVF, and transgender people’s adherence to heteronormative patterns in the context of reproduction) are discussed.

Reflexive thinking was used throughout the research process to reduce unintentional personal bias. We strived to use reflection to increase awareness of our preunderstanding of the study phenomenon in order to minimize any bias of our own influence. Each of us engaged with the other researchers to limit research bias.

#### Ethical considerations

The interviews were conducted in neutral places of the participant’s choice, thereby ensuring privacy and confidentiality and minimizing environmental impact. We adhered to the ethical principles of anonymity, voluntary participation and confidentiality. The participants’ anonymity and confidentiality were maintained throughout the study: to preserve their anonymity, pseudonyms were used to describe participants in this study, and the interviews were registered and stored in a strictly confidential fashion.

## Results

The analysis of the study findings resulted in the identification of the following themes that represent key barriers to pursuing FP or ART: lack of adequate information and fertility counseling, worsening gender dysphoria (fertility treatment may be a challenge to the transition process or a result of it, with the strength of the desire for fertility treatment being crucial), increased discrimination against transgender people due to the rise of extreme far-right populism, low parental self-efficacy, high costs, and the less-than-perfect legal framework. Not all participants expressed a strong desire to have offspring. Various reasons behind transgender people’s desire for parenthood were identified. A number of subthemes were grouped under the base themes, such as the symbolic value of the uterus and pregnancy, the relationship between the type of gender transition and willingness to pursue FP and IVF, and transgender people’s (especially those in social transition) striking adherence to heteronormative patterns in the context of reproduction.

### Lack of fertility counseling

None of the participants reported having received adequate FP counseling before starting their transition, and 6 out of 12 participants indicated that they had not been given adequate information about their FP options.

The participants Jessie (a trans woman who was between 45 and 55 years old and had completed the transition process 2), Luis (a trans man who was between 25 and 35 years old, still in transition), and Jonathan (a trans man who was between 25 and 35 years old, still in transition) did not express regret about the missed opportunity for receiving further information from their psychologist/psychiatrist or endocrinologist about FP. However, the participants Fabiola (a trans woman who was between 18 and 25 years old, at an advanced stage of the transition process), Edward (a trans man who was between 30 and 40 years old, at an advanced stage of the transition process), and Patrick (a trans man who was between 25 and 35 years old and had completed the transition process) made clear complaints about being deprived of the opportunity to make fertility decisions, namely, to have a choice about having children genetically related to them. Furthermore, the participants noted that when they were adolescents in the gender transition, they did not feel ready to make important and lifelong reproductive decisions. However, they were forced to consider whether to preserve their sperm or eggs.

Fabiola, a trans woman who was between 18 and 25 years old, at an advanced stage of the transition process, stated,

*“…A health scientist should have informed me about it... and I went as early as 16... this is what I tell other youngsters, that, ‘OK, you may not be interested in becoming a parent now, but you never know what might happen ten years from now’... no information is given to us...”*

In the same vein, Edward and Jonathan stated that they were not provided with fertility counseling before starting gender transition [for more details, see Additional file 2 (Supplementary Material)].

### Fears of discrimination, bullying, and harassment as barriers to transgender parenthood

- *Bullying by the general population: Discrimination, bullying, and harassment during pregnancy*

The participants expressed fears of discrimination ranging from subtle forms (such as social disapproval) to physical violence. They expressed their fear of aggressive behaviors against them, highlighting the rising extreme far-right populism in the urban areas where they were living.

The fact that the phrase ‘transgender parent’ gives other people a negative impression was reported as discouraging to transgender people with regard to considering FP and assisted reproduction options. Patrick, a trans man who was between 25 and 35 years old and had completed the transition process, said,

*“... it sounds bad... when you say ‘trans parent’, they immediately think, as soon as they hear it, that it is very strange...”*

Fabiola, a trans woman who was between 18 and 25 years old, at an advanced stage of the transition process, highlighting the fear that extreme far-right populist persons may become violent, stated,

*“Imagine a trans man pregnant walking in the town square... to start with, it is dangerous for the person themselves, for their physical integrity…”.*

Fay, a trans woman who was between 45 and 55 years old, still in transition, believed that a transgender parent may be at high risk of bullying by other people as long as she remains visible as a transgender person. However, the participant expressed fears of another form of bullying that may be experienced by transgender parents even if they keep their transgender identity invisible. This form of bullying (the forced removal or separation of children from their parents) occurs in a transgender parent’s family context or is instigated by close relatives [see her quotes below, Additional file 2 (Supplementary Material)].

b) *Bullying by health providers in birth settings*

A trans man who goes to the hospital or a midwifery unit to give birth may commonly be the subject of bullying by health professionals. George, a trans man who was between 55 and 60 years old who had completed the transition process and was bisexual, expressed his fears:

*“The only problem is society, when you go to a maternity clinic with a beard... You will have to be able to go for prenatal birthing classes; you need to receive treatment in an atmosphere of understanding at the hospital, not to be abused.”*

Fabiola, a trans woman who was between 18 and 25 years old, at an advanced stage of the transition process, said,

*“... and how would they be treated during delivery? Does such a person, in other words, have to be rich and go to a private clinic and pay so they are treated with dignity? This does not mean that there are not people in the public health system who do not treat you with dignity [she relates her experience].”*

Unfortunately, health professionals were reported to be the originators of bullying behavior not only within reproductive healthcare contexts but also within other healthcare contexts. Two participants (Fabiola and Edward) described negative experiences with health providers that reflected their providers’ lack of willingness to offer appropriate healthcare to transgender patients. More specifically, they described instances in which health professionals demonstrated subtle (verbal and ‘low-intensity’) bullying-related behavior or at least a lack of empathy for the issues faced [see their quotes below, Additional file 2 (Supplementary Material)].

### The transition process as a barrier to FP and assisted reproduction

- *FP as a challenge for the break with one’s old gender*

Jessie, a trans woman who was between 45 and 55 years old and had completed the transition process, was highly concerned that sperm storage would strongly challenge the (highly desired) break with her old gender identity. She explicitly declared that it would be distressing (for reasons related to gender dysphoria) to pursue FP and explained,

*“... there was no such suggestion by anyone; even if there had been such a discussion, I would not have even stood to hear about it; I wanted to erase any trait left... It is out of the question that I would give my sperm for a biological child... I think this is because it would reduce my female substance (!)… I don’t even remember myself... It’s as if a roller shutter has come down, a curtain, and I cannot see the past... I try to remember me, and I cannot remember me...”*

[In the same vein, the representative quotes of four other participants are presented below, Additional file 2 (Supplementary Material)].

Interestingly, Jessie said that if she had been given the opportunity to undergo uterus transplantation at a younger age, it would have significantly contributed to the success of her transition. As the topic of uterus transplantation was not covered in the interview guide questions, this mention of uterus transplantation came up as an emergent theme. The participant stated,

*“… in other words, it would be continuing on the way to a sense of completion... 100%; I would have felt completed, but, OK, this did not take place when it should have...”*

b) *The highly symbolic value of pregnancy (considered strictly related to femininity) as a barrier to FP and assisted reproduction for individuals undergoing female-to-male gender-affirming transition*

We found that trans men may be very unwilling to become pregnant, whereas they may be willing to become genetic parents.

Antonio, a trans man who was between 35 and 45 years old, still in transition, reported his unwillingness to become pregnant, but he had a strong desire to have children and a family. He was willing to pursue FP and donate oocytes. He stated,

*“ …I am all for having a family and children. Hmmm... if my girl wants to get pregnant, if that is her intention [she is in a wheelchair]; I don’t want to. I want to proceed with the removal, so this will never happen. Any kind of surgery to freeze my ova so that they may be fertilized, if this is possible...”*

Nevertheless, John, a trans man who was between 40 and 50 years old, in social transition, was much more willing to donate gametes (oocytes) than many other participants. Strikingly, he noted that he could not understand why many trans men are not willing to become pregnant, as the desire for parenthood may be stronger than the desire for gender transition. He stated,

*“Yes, absolutely, yes, yes, yes, [I would like to donate an ovum]... this is why, if I am going to receive hormones, I will discuss it a lot with my doctor... after their transition, trans persons do not want to have children as... hmmm... using their body. If you ask me about it, I would say that they would like their boyfriend or girlfriend to do it with another person or to adopt... the question is what [do] you want more: to be a trans person or to be a father? To be a trans person or to be a mother?...”*

Notably, however, some trans men believed that a trans man might become pregnant and give birth after the gender transition. George, a trans man who was between 55 and 60 years old who had completed the transition process and was bisexual, said,

*‘They say that I should have completed the transition and then had children… [If you get pregnant]… the only problem is society, when you go to a maternity clinic…not to be abused’.*

### Placing considerable value on genetic relatedness encourages the willingness of transgender people to become biological parents

The participants reported several reasons for their willingness (or unwillingness) to become biological parents. We remarked on the differences between responses and attitudes related to fertility desire and those related to having children during the interviews. The participants were not always clear about the reasons behind a transgender individual’s willingness or unwillingness to have a child to whom they are genetically related, and the interviewer often needed to ask directly.

The participants in the present study indicated that the desire to have a child to whom they are genetically related has a deeper meaning than just a wish. While rationalizing transgender people’s desire to have a child to whom they are genetically related, the participants discussed several reasons for this desire. For example, Patrick, a trans man who was between 25 and 35 years old and had completed the transition process, placed considerable emphasis on the value of genetic relatedness and biological resemblance between parents and children as the reason behind the desire for biological parenthood and stated,

*“Simply because of the reasons anyone has: that they want to feel it is their own child, made with their own material... to see some features in this child... biological ones.”* [more quotes of Patrick are presented below, Additional file 2 (Supplementary Material)].

In a similar vein, Fay, a trans woman who was between 45 and 55 years old, still in transition, believed that a transgender person’s desire to have children is based on an innate human need to have children and noted,

*“Someone who is a trans individual does not stop wishing they had a child... Just like with cis… I believe that [the wish to have a child] emerges purely from the biological need each individual has.”*

However, this participant thought that the strong desire for parenthood motivates a transgender person to pursue FP techniques and ART and stated,

*“Now, I don’t know if a trans woman would undergo the procedure to have a biological child… only if she truly wants it…” “…I believe things are completely different for homosexuals…”.*

Fabiola, a trans woman who was between 18 and 25 years old, at an advanced stage of the transition process, highlighted that the desire for biological parenthood is egoistically motivated and stated,

*“[I would like a child] for the same selfish reasons any cis person does; I don’t believe [there is] some biological clock... eh, the feeling has to do with selfishness...”*

This view deviated from the dominant culture that highlights essentialism (biology and naturalness). However, on the other hand, the abovementioned participant took a clear stance in favor of biological ties between parents and children. Fabiola missed the opportunity to have her own children (due to a lack of information about FP options before starting her transition) and stated,

*“... what I expect for the future is for my partner to have a child... it would be our child... because this would be my first thought before adoption...”*

Below, Additional file 2 (Supplementary Material) presents more representative quotes of other participants. Jenny strikingly underscored the role of the so-called ‘biological clock’ in shaping the desire for biological parenthood. She was strongly in favor of the natural way of conceiving a baby and strongly rejected the use of medically assisted reproductive techniques. Namely, she remained strikingly steadfast in her adherence to patterns of the dominant culture (based on naturalness/biology and heteronormativity), at least in the context of reproduction. Patrick highlighted the genetic relatedness between parents (more quotes are presented). In contrast, Richard did not emphasize the biological ties between parents and children.

In conclusion, the analysis revealed that transgender people are most likely to have the same basic reproductive needs as cis people, and some transgender individuals place great weight on the value of genetic relatedness.

### Concerns related to transgender parenting and children’s welfare as barriers

- *Transgender people’s fears that their children will be affected by bullying*

Fabiola, a trans woman who was between 18 and 25 years old, at an advanced stage of the transition process, highlighted the social prejudice and discrimination faced by children with transgender parents and stated,

*“…In the local community [reference to the name of the person’s village of origin], even an adopted child is at times pointed to and called a bastard.”*

Interestingly, in the data analysis, fear of social prejudice did not emerge as the main barrier to transgender parenthood related to a child’s welfare.

Surprisingly, Jessie, a trans woman who was between 45 and 55 years old and had completed the transition process, took a clear stance against same-sex parenthood while being in favor of transgender parenthood and said,

*“…I don’t think that we are ready, as a society, let’s say... children are very cruel at such ages and say to another child, ‘I have a daddy and a mummy and you don’t; you have two daddies or two mummies’...”*

b) *Concerns related to the role of the transgender parent (low parental self-efficacy)*

Several participants showed positive attitudes towards transgender parenthood.

Antonio, a trans man who was between 35 and 45 years old, still in transition, said,

*“Whatever love is given, eh... by a straight couple is the same as the love that can be given by a trans person; in essence, eh, love or one’s conduct does not change because of one’s gender identity.”*

However, some participants believed that they would not be able to perform parenting tasks successfully. They were afraid of taking responsibility because they were extra cautious about being responsible for someone else and doing things properly.

Luis, a trans man who was between 25 and 35 years old, still in transition as a pansexual, said,

*“…It’s a very big responsibility to be responsible for someone else...”*

Other participants explicitly expressed their belief that they did not have the qualifications to be a good parent.

Jonathan, a trans man who was between 25 and 35 years old, still in transition, focused on his chronic depression and stated,

*“…I don’t believe that I will ever reach the psychological stage of my life when I am going to want to and be capable of raising a child (psychologically); I suffer from chronic depression, and I don’t know how this may affect a child’s life.”*

Interestingly, some participants were afraid of becoming parents because they were extra cautious about potential dangers to their children (i.e., due to heredity or the toxicity of the use of hormones to embryos or fetuses).

Below, representative quotes of six participants (the aforementioned Luis and five other participants) related to this subtheme are presented [Additional file 2 (Supplementary Material)].

iii) *Concerns about children’s welfare related to a well-established transgender identity*

Some participants felt that gaining a clear gender identity implicitly accepted by others is a prerequisite for becoming a transgender parent.

John, a trans man who was between 40 and 50 years old, in social transition, believed that a transgender individual should gain unambiguous social acceptance of his new gender identity before becoming a parent. The participant stated,

*“[In the past], I did not think of becoming a father, because… there were people who could not accept [my male name], and I had to fight... I believe that trans parents are also parents, but I think that for [a trans person] to start [the process of becoming a parent], everyone must have accepted this... trans person first.”*

The quotes of George related to this subtheme continue below [Additional file 2 (Supplementary Material)].

iv) *Concerns about children’s welfare related to the fact that transgender parenthood diverges from heteronormativity (dominant sexual and gender norms)*

Importantly (though not surprisingly), the participants perceived their adherence to heteronormative patterns of parenting (traditional parent figures) as their motivation for rejecting same-sex and transgender parenthood. Jessie, a trans woman who was between 45 and 55 years old and had completed the transition process, expressed her strong intuition-based prejudice against same-sex parenthood and stated,

*“…I cannot fully ratify this; I may be wrong - should I call myself a racist? I don’t know why, but there is something I don’t like about it; I cannot fully decipher it... I don’t know exactly what it is. Is it being old school?...”*

Jenny, a trans woman who was between 45 and 55 years old, in social transition, placed considerable emphasis on naturalness and explained,

*“The child is going to see me as I am. What can I tell you? If I were in the child’s place, I would like to have a mum and a dad!... Why should I do this? Isn’t it selfish?... It is a sacred thing, Christina!!! It is not only a social issue but also a matter of nature! How can I explain this to you? To your eyes, what is nicer? A photo with mum, dad, grandpa and grandma or a photo with two transvestites? What can I tell you? What seems nicer to you?”*

### High-cost treatments and legal framework as barriers

In this study, economic factors such as the cost of the FP procedure and the storage of gametes were also reported as major barriers to transgender parenthood. For example, the participants Fabiola (a trans woman who was between 18 and 25 years old, at an advanced stage of the transition process), Edward (a trans man who was between 30 and 40 years old, at an advanced stage of the transition process), Luis (a trans man who was between 25 and 35 years old, still in transition as a pansexual), and Jenny (a trans woman who was between 45 and 55 years old, in social transition) highlighted this barrier. Moreover, Jessie (a trans woman who was between 45 and 55 years old who had completed the transition process), and Fabiola, (a trans woman who was between 18 and 25 years old, at an advanced stage of the transition process), reported the perceived as less-than-perfect Greek legal framework (as anticipated above) as a barrier to transgender parenthood.

## Discussion

### Lack of adequate fertility counseling

One of the problems that transgender people often face related to FP and assisted reproduction is the lack of information. Consistent with past literature, our study findings showed that a significant barrier to pursuing FP and/or assisted reproductive techniques was the lack of counseling about FP options.

Over the last decade, many authors have highlighted the need for vulnerable populations of transgender adolescents and young adults to be provided with fertility counseling prior to the initiation of the gender-affirming care process [8, 9, 11, 34–36] 3. Fertility counseling should be highly prioritized as an ethical, interdisciplinary practice [37–41]. Despite multiple papers being written about the need for this issue to be addressed, almost all the participants in this study felt that FP had not been adequately offered. Previous literature has highlighted that transgender people should be provided with ‘enough information, support and opportunity to make an informed decision about fertility preservation’ [8] 4. Very recently, Sterling and Garcia (2020) argued that a ‘lack of reliable information available from other and outside sources’ is among the most common reasons for the discrepancy between reported high interest in FP and a very low utilization rate [20] 5. The authors stressed that physicians need ‘better *training* about transgender patients in general, and FP options available to them’ [20]. Petit, Julien & Chamberland (2018) also stated that physicians must be trained to be aware of transgender persons’ specific challenges and to better support them [15].

Moreover, it should be highlighted that some children/pubertal children/adolescents/young adults may not yet be mature and competent enough to evaluate, on their own, whether to pursue FP [37]. Some of the participants described a point at which they should have received information about FP options, even though, due to being a minor at that time, they might have been unable to fully understand either the implications of their reproductive decisions or their future attitudes towards having biological offspring many years later in their lives. This was the case with some participants (Fabiola and Edward). In such scenarios, questions may arise regarding decision-making authority [11].

### Barriers related to discrimination and bullying

Barriers related to discrimination and bullying were one of the frequent themes and encompassed the subthemes of bullying during pregnancy by the general population and bullying by health professionals in birth settings [42]. Across the globe, transgender people are extremely vulnerable to physical and sexual violence and experience epidemic levels of stigma, discrimination, harassment and social rejection in almost every aspect of their daily lives, including their access to health care services [43–46] 6. Being a transgender parent is still heavily stigmatized in Greece. Kantsa (2014) argued that ‘normative concepts of kinship …are acquired through a heterosexual marriage ‘blessed’ with children’ [27]. In addition, the rise of extreme right-wing populism (due to economic crises in both urban and rural areas) that is openly violent and racist seems to be a theme in the Greek political scene [47, 48] 7.

Our analysis showed that stigma against pregnant trans men can occur in hospitals or midwifery units where pregnant trans men have to go to give birth. This finding is consistent with previous research. Societal attitudes ‘erect barriers to openly being pregnant and giving birth as a transgender man’ [13] 8. Charter et al. (2018) stated that ‘healthcare systems are not generally supportive of trans bodies and identities and trans men encounter significant issues when interacting with healthcare providers’ [12]. This is consistent with many other studies [13, 18, 41, 49]. There are institutional barriers to transgender men receiving routine patient-centered perinatal healthcare services [13]. Trans men who are gestational parents ‘seek to normalize their experiences of conception, while also acknowledging the specific challenges they face’ [18]. Furthermore, Armuand et al. (2020) found that physicians said that they ‘had little knowledge about the next step following FP as they only had vague knowledge about the transgender men’s reproductive choices and legal rights’ [42].

Regarding Greece, Giannou (2017) reported that in Greece, transgender people often experience discrimination by healthcare providers, ranging from disrespect or transphobic insults to outright denial of service, when accessing healthcare services [24]. This discrimination can be seen as a public health issue. Notably, Armuand et al. (2020) found that health care professionals ‘experienced important challenges to their professionalism when their preconceived opinions and values about gender and transgender were confronted’ [42]. Such challenges may contribute to an unsafe environment for transgender people undergoing FP through various procedures, which may heighten their distress. Health professionals should manage to rethink communication and maintain professionalism when encountering transgender people [42].

Importantly, according to the narratives of the participants in our study, this prejudice was going ‘underground’ and was being expressed in more subtle, indirect ways. This is not surprising, given the truth of the assumption that anti-homosexual prejudice is no longer exercised in the traditional, ‘old-fashioned’ form (openly related to adherence to ‘naturalness’) but rather in a modern, subtle, ‘nondiscriminative’ form [50]. Furthermore, in the context of Greece, there may be an additional explanation for this phenomenon. Being a transgender person is stigmatized in Greece, a traditionally conservative country. However, recently, attitudes towards transgender people have become somewhat more positive. Because Law n. 4491/2017 allows citizens to choose to legally change their gender identity (from the age of 15), policy and *public opinion have given increased attention to transgender people* during the last few years. At any rate, the findings related to discrimination and bullying by health professionals call for efforts by the health service system to provide equal access to fertility and reproductive health services for transgender people. Armuand et al. (2017) argued that health professionals can ‘alleviate distress by using gender-neutral language and the preferred pronoun’ [51]*.* Riggs & Bartholomaeus (2020) highlighted the need for ‘the continued development of trans reproductive justice’ [16].

### FP and/or IVF may worsen dysphoria and delay effective transitioning

The impact of FP and/or IVF on the worsening of dysphoria and the delay of effective transitioning was a significant theme. Consistent with past literature, we found that among transgender people, there are unique barriers to FP related to gender dysphoria. Transgender adolescents face several obstacles that affect fertility decision-making [36, 37], including the invasiveness of procedures, individual experiences of gender dysphoria, and a desire not to delay the gender-affirming transition [36, 52]. De Sutter et al. (2002) found that while the vast majority of respondents thought that FP should be offered to transgender women, 90% of respondents believed that the loss of fertility was not a strong reason to delay the transition [53]. This is consistent with the statement of Chiniara et al. (2019) presented below in footnote [54].

Chen and Simons (2018) effectively explained that ‘transgender adolescents pursuing hormones may be at particularly high risk for prioritizing short- versus long-term outcomes, putting them in jeopardy for later experiencing regret’ [6]. Importantly, FP methods ‘might reinforce transgenders’ previous sex or make them feel it does not fit with their new gender identity’ [11]. Interestingly, procedures required for FP (i.e., hormonal ovarian stimulation and transvaginal ultrasound, which is a genitalia-specific procedure) may be experienced by trans men as having the negative impact of worsening their gender dysphoria [51]. These procedures may heighten feelings of dysphoria, thus challenging transgender people’s break with their old gender identity. Note, however, that this is not always the case [51].

This may partly explain the reluctance of trans men to become pregnant. Nahata et al. (2017) argued that ‘more research is needed to understand parenthood goals among transgender youth at different ages and developmental stages and to explore the impact of gender dysphoria on decision-making about FP and parenthood’ [55]. This was a significant theme that emerged from our data analysis because there were a large number of comments related to this category, and considerable emphasis was placed on this topic by the participants in our study. However, it is crucial to bear in mind that ‘presently little is known about the psychological effects of FP for transsexuals’ [11], and the number of related studies is still limited.

Furthermore, transgender people’s break with their old gender identity may be challenged by the fact that it cannot be ruled out that future children will be informed about their parent(s)’ status as transgender persons [11]. It is noteworthy to mention that none of the participants raised concerns about such problems.

Moreover, we found that the highly symbolic value of pregnancy is likely a barrier to FP and assisted reproduction for individuals undergoing female-to-male gender-affirming transitions. Given that pregnancy is considered to be strictly related to femininity, it may negatively affect a trans man’s gender transition by challenging his break with the old (female) gender identity. However, this is not always the case. It is argued that trans men use contraception and can experience pregnancy, even after having transitioned socially, medically, or both [14, 56]. Moreover, notably, one participant explained that the symbolic value of the uterus may effectively facilitate the gender tradition process, most likely based on the common acceptance that pregnancy is a women’s affair and strongly related to femininity. The participant said that a uterus transplant at a younger age (if possible) would make her feel 100% a woman. The phenomenologist Svenaeus (2012), analyzing the changes in identity and selfhood experienced through organ transplantation, stated that ‘…the organ in question is taken to harbor the identity of another person, because of its symbolic qualities…’ [57] 9. Not surprisingly, a trans woman may desire to have the woman-specific experience of gestation. However, such a right might be controversial [58, 59].

Trans men may be more likely to become parents after gender transition [7]. Some transgender men retain their uterus [13]. Some participants (trans men) were explicitly willing to donate their oocytes and become genetic parents. Interestingly, we found that a trans man in social transition was much more willing to donate gametes (oocytes) than many other participants. His strong willingness may have been partly because he was not in a gender-affirming transition but in a social transition. Many trans men participants in our study touched upon some aspect of oocyte cryopreservation. It is of great importance that little is known about transgender men’s experience with FP procedures such as cryopreservation of oocytes due to a lack of previous empirical research on this topic [51]. Recently, a study found that ‘adolescent transgender males who choose to undergo oocyte cryopreservation tolerate the process well’ [60]. The aforementioned findings of our study gave us the opportunity to formulate starting points for further research. These points are presented below in the section ‘Implications for practice and further research’.

### Placing considerable value on genetic relatedness motivates transgender people to have the willingness to become biological parents

Involuntary childlessness is associated with serious negative psychological effects: serious anxiety and stress, feelings of grief, social isolation, low self-esteem, and sexual dysfunction [61–63]. Furthermore, according to a holistic positive concept of health, involuntary childlessness can be regarded as an unhealthy situation.

Reproductive desire was high among the majority of the participants in the present study. Notably, however, this view may be a result of mechanisms such as ex post realization or the overgeneralization of hard-wired perceptions due to low self-esteem (which, in turn, may be due to internalized anti-trans prejudice). Further studies are needed to assess whether internalized anti-trans prejudice is associated with a weak desire among transgender people to have a child to whom they are genetically related or to an unwillingness to have children.

Prior studies have suggested that reproductive desire is as high among transgender people as it is in the general population [35, 40, 53]. Among transgender adolescents, the utilization rates of FP and reproductive options are currently very low [10, 55] but steadily rising [11, 35]. In 2012, it was argued that ‘research on transgender adults suggests that about half desire biological children…, and over a third would have considered FP had such technologies been available at the time of their transition’ [35]. In our small sample, this percentage was much greater. The lack of adequate FP counseling may partly explain these low rates [10]. Riggs and Bartholomaeus (2018) argued that FP should be made available to all transgender people before they undergo gender transition treatment that could negatively affect their future fertility, although *not all transgender persons would be willing* to undertake FP [17]. Nevertheless, this topic seems to be complex [54, 55, 64, 65] 10.

### Barriers related to parenting and the child’s welfare

Barriers related to parenting and the child’s welfare were a frequently recurring theme in our interview data analysis, and several participants in our study identified such barriers. There were various types of reported barriers, and they can be categorized into the following three subthemes:

- *Barriers related to the social environment (prejudice against children of transgender parents)*

Transgender people’s children are vulnerable to discrimination and bullying. Although the best currently available evidence does not support the notion that there are inherent risks to the welfare of the child of a transgender person, there may be external risks to the welfare of the child based on social discrimination and stigma [11]. Having children is strongly related to heteronormative stereotypes.

b) *Barriers related to transgender parents’ perceived limited parenting capability*

The majority of the participants in our study felt incapable of meeting the standards of adequate parenting or perceived themselves as potentially harmful to their children. From the analysis of their statements and their corresponding nonverbal behavior patterns, we sensed that they drew unfair conclusions about their parental capacity based on low self-esteem. Internalized transphobia may negatively impact self-esteem [66] and hence limit transgender people’s (reproductive) autonomy [67], and this may be the real reason behind the unwillingness of transgender people to become parents. Transgender individuals’ parental role is a complex issue. Petit et al. (2017) stated that ‘…trans parental identity appeared as a multidimensional, multidetermined, nonbinary, and fluid identity in a context of nonalignment between the sex assigned at birth and gender identity’ [68]. This nonalignment may heighten feelings of parental incapacity.

iii) *Barriers related to transgender individuals’ values (adherence to patterns of the dominant culture)*

According to the findings of the present study, transgender individuals may have both new and old understandings of patterns related to parenthood, such as biological relatedness and parenting figures. This finding is consistent with past literature on issues of LGBT parenthood [69].

In conclusion, several of the aforementioned findings in the ‘Concerns related to transgender parenting and children’s welfare as barriers’ section of the paper suggest that some transgender people have very low expectations about what kind of parents they would become; that is, they have low parental self-efficacy. Moreover, it is worth noting that we identified several subthemes grouped under the theme ‘concerns related to child welfare’. In our opinion, the presence of several subthemes for this supports the assumption that transgender parenthood is a complex, complicated, and multidimensional issue.

### Barriers related to economic instability

The costs of FP are a significant barrier because these procedures are typically not covered by insurance companies [37]. Transgender people are particularly vulnerable to economic instability due to their high unemployment rate related to the mere fact of being transgender. Riggs and Bartholomaeus (2018) argued that while ‘fertility preservation should be made available as an option to all transgender or nonbinary people prior to undertaking treatment which may impact on fertility’, ‘not all people may be able to afford to’ [17]. Very recently, Sterling and Garcia (2020) suggested that ‘the considerable out-of-pocket costs’ may be one of the common reasons why, despite a reported high level of interest among transgender persons in FP, there was a very low utilization rate [20]. Furthermore, it should be noted that there are still high unemployment rates in Greece due to the Greek financial crisis.

### Strengths and limitations

This research is important in that to our knowledge, it is the first to directly examine transgender people’s attitudes towards the use of FP options or assisted reproductive techniques in Greece.

However, our study has two primary limitations. First, our findings cannot readily be generalized to larger populations because of the small number of participants. However, the findings of this study might be applicable to other transgender people. While qualitative studies may sometimes be criticized for their limited generalizability due to small samples, in our opinion, they remain valuable as indicators of the range of views within the public and how these views may be influenced. Second, the participants in this study reflected on their past experiences, which, for some, had occurred more than 10 years prior to being interviewed. Recall bias may have distorted the recollections of their experiences considering FP options or assisted reproductive techniques. To minimize recall bias, we attempted to establish a climate that would enable the participants to recall their lived experiences and events that occurred many years before as it related to having a child to whom they are genetically related and to pursuing fertility treatments. Moreover, we spent more time with older participants to help them return to their youth when they had reproductive options. We provide more details in the limitations section.

### Implications for practice and further research

The results of our analysis of the study data may have implications for both research and clinical practice. These results could provide guidance for professionals processing transgender people’s applications for medically assisted reproduction and FP. We highlight the need for training for health professionals to establish a safe environment for transgender people who are willing to pursue FP or IVF, especially in places (in both urban and rural areas) where there is a high prevalence of extreme right-wing populism in the context of the Greek economic crisis.

Moreover, we emphasize that rigorous psychological evaluation is required. Careful, in-depth psychological evaluation would provide important information for understanding the primary reason behind a transgender individual’s attitude towards fertility matters. In the short time frame of the interview, Patrick, a trans man who was between 25 and 35 years old and had completed the transition process, reported four reasons for his unwillingness to consider FP options or assisted reproductive techniques. The participant provided a basis for the assumption that these reasons (mentioned elsewhere in this paper) are equally strong. For instance, the participant’s attitude might have resulted from mechanisms such as ex post realization or overgeneralization of hard-wired perceptions.

At any rate, our findings could heighten awareness of and stimulate debates about the ethical topics related to our research questions.

Furthermore, based on the findings of our study, we provide some starting points for further research. For instance, the association between the type of transition and the willingness to become involved in procreation remains to be tested. Moreover, it remains to be further explored whether transgender individuals who are in social transition show greater adherence to the dominant culture than those in gender-affirming transition, at least in the context of reproduction. Last, we stress the need for further empirical research into transgender men’s experience of FP procedures such as the cryopreservation of oocytes. In this vein, it would be interesting to investigate whether transgender people should be classified as a separate group of the LGBT community and whether data on transgender individuals should be analyzed separately.

## Conclusion

The results demonstrate the importance of both contextual factors (stigma, economic instability, and law) and factors related to transgender people themselves (gender dysphoria, the desire to become parents, and self-trust). More specifically, the conducted analysis resulted in the identification of the following themes that represent key barriers to pursuing FP or ART: lack of fertility counseling; high costs and economic instability (due to the Greek economic crisis); concerns related to the child’s welfare due to factors related to the context or transgender people themselves; a less-than-perfect legal framework on transgender people’s rights; concerns about whether fertility treatment may negatively impact the gender transition process; fears of discrimination (by the general population or even health care providers); and fears of bullying in the traditionally conservative Greek societal system, which embraces heteronormativity and is gradually emerging from a decade-long economic crisis that gave rise to extreme far-right populism. A number of subthemes were grouped under the primary themes. Various reasons behind the transgender participants’ varying degrees of desire for parenthood were identified. Furthermore, the results indicated the symbolic role of the uterus (important to trans women) and pregnancy-related body changes (important to trans men, as they act as a barrier to the gender transition process and give rise to discrimination against them). The results allowed us to hypothesize that transgender individuals in social transition are much more willing to pursue FP or ART (or, for trans men, to become pregnant) than those in gender-affirming transition. In addition, transgender individuals showed striking adherence to patterns of the dominant culture in regard to attitudes towards having children and low self-esteem.

Transgender people’s willingness to pursue FP and/or IVF is a complex topic, and we highlight the need for rigorous individual psychological evaluation. Moreover, we stress the need to train health professionals to establish a safe environment for transgender people who want to undergo fertility treatment, become pregnant and give birth. Health professionals should be trained to develop trans reproductive justice.

The findings of this study call for efforts by the fertility and reproductive health service system to support and provide equal access to fertility and reproduction-related services for transgender people. Addressing the barriers to transgender parenthood that are documented in this article will require policy initiatives and a social justice approach towards transgender individuals’ health and human rights. Health providers can play a crucial role in this process. Therefore, the need to establish standardized protocols and provide necessary training to physicians is highlighted.

## Supplementary Information

**Additional file 1.**

**Additional file 2.**

## Data Availability

The transcripts of the full interviews that were collected and qualitatively analyzed in the current study are not available due to the ease with which study participants could be identified. The redacted transcripts used and analyzed during the current study can be made available by the corresponding author on reasonable request.

## Abbreviations

FP: Fertility preservation
ART: Assisted reproductive technology
IVF: In vitro fertilization

## Footnotes

1 The term ‘transition’ is used to refer to all types of gender-affirming (endocrine or surgical) transitions. The term ‘social transition’ is used to specifically reflect gender transition. The participants in ‘social transition’ opted not to undergo gender-affirming treatment, believing that a change in their gender role or behavior would itself be sufficient.

2 At the time of the interview, ‘still being in transition’ was ‘being on the road to what the particular participant *perceived* as full transition’.

3 In 2012, Wierckx et al. remarked that transgender people’s fertility issues were not adequately addressed [35]. This observation still applies in the present day. Chen et al. (2019) found shortcomings in fertility counseling and providers who highlighted the need for standardized counseling protocols [36]. Interestingly, their findings indicated that transgender people could later regret not pursuing FP despite having previously received FP counseling.

4 Murphy (2012) argued that there is nothing objectionable that would justify removing parenting options for transgender people [39]. The American Society for Reproductive Medicine (ASRM, 2015) stated that “transgender persons have the same interests as other persons in having children” and that “providers should offer FP options to individuals before gender transition” [40]. The Ethics Committee of the American Society for Reproductive Medicine stated that transgender people’s gender identity cannot be grounds for unequal treatment and that professional autonomy is not a sufficiently strong countervailing reason to justify an exemption. Transgender people should be provided with ‘enough information, support and opportunity to make an informed decision about fertility preservation’, and the discussion should include ‘a consideration of interweaving factors, particularly costs…’ [8]. ‘Detailed information about every option in the absence of any form of coercion and with ample time is essential for a person to make complex, life-changing decisions’. [38]. The importance of genetic relatedness might be used as a ‘heuristic through which to provide fertility counseling to transgender people’ [41]. From the perspective of transgender people’s fertility counseling, health professionals communicate with transgender people about desires related to reproduction [9]. Furthermore, transgender people should be informed that ‘FP methods do not guarantee future access to medically assisted reproduction (due to the best evidence then available, i.e., concerning the child’s welfare) or successful reproduction’ [11].

5 Notably, however, that discrepancy may (partly) be because physicians feel most uncomfortable talking about transgender planned parenthood. Sterling and Garcia (2020) argued, ‘Transgender patients report using assistive reproductive services difficult, due to a lack of dialogue about fertility and the lack of information offered to them- presumably because their circumstances do not fit into a traditional narrative familiar to providers’ [20].

6 In Europe, the European Union Agency for Fundamental Rights (2014) reported that approximately 20% of all trans respondents who accessed healthcare services or social services reported that they had experienced discrimination for the same reason [43]. In Australia, although in 2013 the Sex Discrimination Act was amended, transgender individuals still experience discrimination and barriers to access to health care services [44]. Much of the same holds for Asia [45] as well as for Latin America and the Caribbean [46].

7 Therefore, according to many participants, the fear of violence against pregnant trans men or transgender parents was greater in urban areas (with a high percentage of extreme right-wing populism), although normally urban areas are more tolerant, open-minded, multicultural and less conservative and traditional than small towns or villages (provinces or rural areas).

8 A Canadian interview study found that transgender men face considerable discrimination throughout their pregnancy [49]. Riggs (2013) found that transgender men who go through a pregnancy negotiate complex intersections between their masculinity and child bearing, with their pregnant bodies being regarded by health care providers as female [41].

9 Notably, however, Robertson (2017) argued that procreative liberty only supports a right to gestate when gestation is sought for genetic reproduction, and hence, the claim of a transgender woman desiring a uterus transplant to have the woman-specific experience of gestation is not strong enough to undergird a positive right [58]. Notwithstanding, Alghrani (2018) argued that procreative liberty does extend to a right to gestate [59].

10 A U.S. study found that only two of 72 transgender young people receiving fertility counseling prior to endocrine transition attempted FP [55], while a recent study with a Dutch cohort of trans girls found a much greater percentage attempting FP [64]. Persky et al. (2020) found that the majority of transgender youth were not willing to delay their hormonal transition for FP, as they ‘did not find having biological offspring important’ [65]. Chiniara et al. (2019) arguably hypothesized that fertility may be a low life priority for young transgender people. ‘The majority wish to become parents but are open to alternative strategies for building a family’ [54].
