# Supplementary material for: A qualitative study examining transgender people’s attitudes towards having a child to whom they are genetically related and pursuing fertility treatments in Greece
Source: BMC Public Health. 2021 Feb 18;21:378. doi: 10.1186/s12889-021-10422-7 (PMC7890100; doi:10.1186/s12889-021-10422-7)
Supplement: Supplementary file 1 — Additional file 1. [file 12889_2021_10422_MOESM1_ESM.docx]

**Additional file 1**

**Interview Guide**

1. What is it like to be a transgender parent, and what does it mean you? (*a grand tour question to make the participant comfortable*).
2. How do you think other transgender people perceive having a child to whom they are genetically related?
3. What would motivate or did motivate you to pursue or not pursue parenthood?
4. What do you know about other transgender people’s experiences or attitudes towards pursuing fertility preservation or in vitro fertilization techniques?
5. What are your lived experiences or attitudes towards pursuing fertility preservation or in vitro fertilization techniques?
6. Can you please describe in detail what types of barriers a transgender person needs to overcome to pursue fertility preservation or in vitro fertilization techniques?
7. Can you please describe in detail what kind of concerns might be associated with transgender parenthood (achieved through medically assisted reproduction)?
8. Please give me as many details as you can remember about your personal experiences (if any) with transgender parenthood and striving to achieve it.
9. What do you perceive to be the most important problems and challenges that transgender persons encounter in striving to achieve biological parenthood?
10. Of all that we have discussed, what is the most important issue in your opinion?
11. Is there anything else anyone would like to bring up?
