# Supplementary material for: A qualitative study examining transgender people’s attitudes towards having a child to whom they are genetically related and pursuing fertility treatments in Greece
Source: BMC Public Health. 2021 Feb 18;21:378. doi: 10.1186/s12889-021-10422-7 (PMC7890100; doi:10.1186/s12889-021-10422-7)
Supplement: Supplementary file 2 — Additional file 2. [file 12889_2021_10422_MOESM2_ESM.docx]

**Additional file 2**

**Results (Supplementary)**

**Lack of fertility counseling**

Edward, a trans man who was between 30-40 years old, at an advanced stage of the transition process, said,

*"...if I had known when I was 20 [about cryopreservation], I don't know what I might have done. Some people did not make this choice because they did not know about such an option, and they might have wanted to make such a choice later on…"*

Jonathan, a trans man who was between 25-35 years old, still in transition, stated that he was not provided with fertility counseling before starting gender transition because, in the healthcare context, he came across as being uninterested in having children. Reflecting on his experience, he said,

*"They did not talk about this; it was not their priority for any reason... in the health system... They knew that this matter did not concern me…"*

**Fears of discrimination, bullying, and harassment as barriers to transgender parenthood**

1. ***Bullying by the general population: Discrimination, bullying, and harassment during pregnancy***

Fay, a trans woman who was between 45-55 years old, still in transition, stated,

*"Now, look! If you see a trans person in public who shows they are trans, if they go out with the child, they may be taunted, they may have to face many things, I believe. If it does not show, I believe they will not face any particular problem, unless there is a problem in their environment, their closer family circle... the [family members] may set procedures in motion to take the child themselves or send him/her [the child] to an institution or something. All that matters is that the child should not be with the trans individual, which is the worst thing for them..."*

1. ***Bullying by health providers in birth settings***

Fabiola, a trans woman who was between 18-25 years old, at an advanced stage of the transition process, recalled,

*"… When I visited a plastic surgeon for breasts, he had forgotten my problem; he was, like, 'Is a psychiatrist attending to you? Are you seeing any doctor? What kind of hormones have you taken? What other operations?' I felt, in a way, [that] I was being abused. Because there were other people present..."*

In a similar vein, Edward, a trans man who was between 30-40 years old, at an advanced stage of the transition process, detailed his experience:

*"… access to the health sector is very difficult for us... and an unpleasant experience, right? How can you go to the hospital and hear them ask you, 'Now, what are you?' 'What is it that you've got under your knickers?'..."*

*… [the health professional] hardly looked at my health booklet, although I had explained that I was a transexual person... He took one look at the injection, and he went, 'Ah... testosterone... why are you having this shot? ', in front of other people, and I go, 'I am going to tell you'..."*

**The transition process as a barrier to FP and assisted reproduction**

1. ***FP as a challenge for the break with one’s old gender***

Luis, a pansexual trans man who was between 25-35 years old, still in transition, expressed strong concerns about worsening his gender dysphoria by completing invasive FP (at least without strong countervailing reasons). He believed that going through the FP procedure (i.e., hormonal stimulation and egg retrieval) could be quite invasive and noted,

*"… I am not going to subject my body [to this] and risk my mental health and serenity if it is not absolutely necessary... I don't know how it might affect my emotions because I am trying to break free from that gender; I would not like to go back to such symptoms..."*

Jonathan, a trans man who was between 25-35 years old, still in transition, made it clear that it could be distressing (have a negative impact on gender dysphoria) to delay gender transition to facilitate FP or to undergo invasive FP procedures while having to wait for the gender-affirming care to start.

*"I was thinking about doing this before I started the transition, but the procedure was truly difficult even before the transition because of the hormonal disorders... No way could I have had the physical or mental strength to put up with this; that's why I am looking forward to my hysterectomy, to be done with this matter once and for all."*

Furthermore, oocyte storage may challenge the break with the transgender individual’s old gender identity, even if the individual has completed the transition process. Patrick, a trans man who was between 25-35 years old and had completed the transition process, stated,

*"... I think it is difficult to communicate this to the other person... to sit and tell him, 'You know, I have some [ova] stored ... and we can do it this way'... I don't know how easy that might be."*

Fabiola, a trans woman who was between 18-25 years old, at an advanced stage of the (endocrine) transition process, discussed her worry that treatment with testosterone to improve sperm quality would significantly challenge her process of gender-affirming transition and, hence, that the effort would not be worth it, as the success rate is very low and there is therefore no strong reason for doing it. She explained,

*‘A child of my own? I don't think this is possible anymore... because I have no intention to reverse my hormone treatment, so I am telling you, wittingly, THIS possibility is out of the question for me, i.e., to become a biological parent; I do NOT exclude becoming a parent, but I DO exclude the biological aspect of it. Because I would have to reverse the hormonal treatment, which I am not going to do... why should I give testosterone to my body? Whatever for? For something that is very unlikely to be successful? Because the chances they give you that my sperm will be OK are very low... This would take me way back in time, for my appearance as well..."*

1. ***The highly symbolic value of pregnancy (considered strictly related to femininity) as a barrier to FP and assisted reproduction for individuals undergoing female-to-male gender-affirming transition***

**Placing considerable value on genetic relatedness motivates transgender people to have the willingness to become biological parents**

Jenny, a trans woman who was between 45-55 years old, in social transition, strikingly underscored the role of the so-called ‘biological clock’ in shaping the desire for biological parenthood and stated,

*"Whether you are a trans-sexual or a bisexual or a heterosexual, aren't you going to have a kid? Therefore, don't you want to have a family and a home for this child?… You are beautiful yourself, why adopt? [Having a child] is a blessing from nature... For better or worse, when the biological clock ticks, everyone wants a child..."*

The aforementioned participant, Jenny, was strongly in favor of the natural way of conceiving a baby. She rejected the use of medically assisted reproductive techniques, saying,

*"Artificial insemination/cryopreservation? I really don't want any of all this, dear girl! In other words, I prefer more traditional things. Even a lesbian who wants to have a child could find a one-night stand and have a child… Frozen sperm? Yuck! Not for me!"*

Surprisingly, this participant remained strikingly steadfast in her adherence to patterns of the dominant culture (based on naturalness/biology and heteronormativity), at least in the context of reproduction.

It is worth mentioning that Patrick, a trans man who was between 25-35 years old and had completed the transition process, highlighted the genetic relatedness between parents and children and conveyed the impression that if he had ‘excellent DNA’, it would constitute a strong reason for making him willing to pursue FP and donate oocytes to his partner. He stated,

*"…Personally, I couldn't care less if the child is mine; ha, ha, OK [to donate, e.g., ova to his girlfriend to get pregnant]; I don't even believe that my DNA is anything special... so, this is what I believe."*

Not surprisingly, Richard, a trans man who was between 35-45 years old, in the final (almost complete) stage of the transition process, did not emphasize the biological ties between parents and children. Interestingly, he believed that genetic and social parenthood should be thought of as having equal value, while considerable emphasis should be placed on values such as love and affection between parents and children. This view clearly deviated from the essentialist reasoning regarding parenthood that highlights nature (biology), which is strictly associated with the dominant culture and ideology. The participant stated,

*"Sharing ova [giving one of his to his partner]? Hm, no... what I mean is, won't it be my child if I raise it? Is it necessary for the child to have my ova so that it is mine? The point is, if you have a child, whether biological or not, you have to love it. In other words, if it is not your biological child, you are not going to love it?"*

**Concerns related to transgender parenting and children’s welfare as barriers**

***a) Transgender people’s fears that their children will be affected by bullying***

***b) Concerns related to the role of the transgender parent (low parental self-efficacy)***

Some participants did not raise concerns about their possible role as parents.

George, a trans man who was between 55-60 years old who had completed the transition process and was bisexual, said,

*"…gender identity has nothing to do with wanting to have a child."*

In the same vein, Edward, a trans man who was between 30-40 years old, at an advanced stage of the transition process, noted,

*"...Everyone is entitled to becom[e] a parent; what is necessary is for relevant legislation to be in place, as we said; what is necessary is to study the situation so some things are done correctly..."*

Similarly, Jessie, a trans woman who was between 45-55 years old who had completed the transition process, said,

*"This has nothing to do with gender; [both trans and cis] should have [a child], why not? They have love to offer, and many other things that everyone can give..."*

Some other participants raised concerns about their possible role as parents.

Jessie focused on her characteristics and added to her aforementioned quotes,

*"…I think I would be overprotective and possibly authoritarian; I might not be able to fully control and fully manage that..."*

Luis, a trans man who was between 25-35 years old, still in transition as a pansexual stated,

*"… I am bipolar, OK? I don't know if it is passed down, if it is hereditary..."*

*"... but, if my child told my 'Dad, I am trans'... I would not like the child to be subjected to the procedure I have been through..."*

While Fay, a trans woman who was between 45-55 years old, still in transition, believed that a child raised by LGBT parents would receive ample love and affection, she feared that the parent’s hormone replacement therapy might negatively affect the health of the child.

*"If you have taken hormones, then the child may be born with problems, which means it would have been better not to have had it... why bring a child with problems into the world, to suffer?"*

Patrick, a trans man who was between 25-35 years old and had completed the transition process, said,

*"... [I would like] if something goes wrong, for example, that the child should be more my girlfriend's... but I think I generally prefer adoption."*

1. ***Concerns about a child’s welfare related to a well-established transgender identity***

George, a trans man who was between 55-60 years old who had completed the transition process and was bisexual, noted,

*"… First of all, you need to feel OK with who you are, to know who you are and where you are going and then [have a child] … They say that I should have completed the transition and then have children... And now, sometimes, they call me 'mamo'; my daughter [tells] her fiancé, 'My mother is not like others, she is a trans man; this is how we live'..."*

1. ***Concerns about children’s welfare related to the fact that transgender parenthood diverges from heteronormativity (dominant sexual and gender norms)***

**High-cost treatments and legal framework as barriers**
